# Peimine Promotes Skin Wound Repair in Mice by Activating the Notch1 Signaling Pathway in Fibroblasts

**DOI:** 10.1002/fsn3.70406

**Published:** 2025-06-08

**Authors:** Peng Wu, Cheng Zhang, Congcong Wu, Junzhe Lang, Lili He

**Affiliations:** ^1^ Department of Orthopaedics The First Affiliated Hospital of Wenzhou Medical University Wenzhou China; ^2^ Department of Dermatology The First Affiliated Hospital of Wenzhou Medical University Wenzhou China

**Keywords:** healing rate, Notch1, peimine, vascular regeneration, wound healing

## Abstract

This study aimed to investigate the effects of peimine on cutaneous wound healing efficiency and tissue regeneration in murine models, while exploring its regulatory mechanisms through the Notch1 signaling pathway. Full‐thickness circular skin defects (8‐mm diameter) were surgically created on the dorsal surface of C57BL/6 mice, with subsequent daily topical administration of peimine at 1 and 4 mg · kg^−1^ doses. Parallel in vitro experiments using NIH/3T3 fibroblasts were conducted with peimine treatments at 25 and 100 μmol · L^−1^ concentrations to assess neurogenic locus notch homolog protein 1 (Notch1) pathway activation. Key findings demonstrated that peimine treatment significantly enhanced both the rate of wound closure and granulation tissue formation. Histological analysis revealed increased epidermal thickness in peimine‐treated groups compared to controls. The compound promoted extracellular matrix remodeling in the dermal layer, evidenced by elevated protein expression of collagen IIIα1 (Col3α1), proliferating cell nuclear antigen (PCNA), and the endothelial marker (CD31). Western blot analysis confirmed consistent upregulation of Notch1 pathway components in both in vivo wound tissues and in vitro fibroblast cultures, indicating that peimine accelerates wound repair through Notch1 signaling activation.

## Graphical abstract

This study clarifies the potential therapeutic effect of peimine on promoting skin wound repair, which might benefit from accelerated granulation tissue formation, epithelial regeneration, collagen deposition, proliferation, and vascular regeneration via regulating the Notch1 signaling pathway on fibroblasts in mice. It provides new insights for the development of peimine and directing its clinical application.

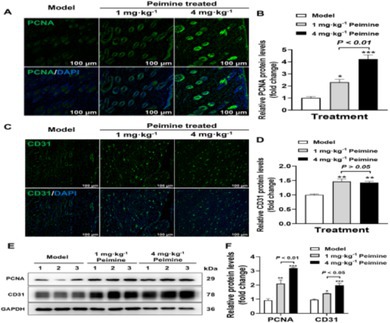

## 1 Introduction

Skin wound healing is a complex biological process mediated by cytokines, extracellular matrix (ECM) dynamics, and coordinated intercellular communication (Jiang et al. 2023; Peña and Martin 2024; Talbott et al. 2022). This process progresses through three distinct phases (1) initial hemostasis and inflammatory response activation, (2) intermediate proliferation phase characterized by keratinocyte/fibroblast migration, angiogenesis, and matrix deposition, and (3) final remodeling stage involving tissue maturation and re‐epithelialization.

The neurogenic locus notch homolog protein (Notch) signaling pathway critically regulates cutaneous development and repair mechanisms (Gratton et al. 2020; Serrano‐Coll et al. 2020). Notch signaling, specifically the Notch1 axis, promotes sebocyte differentiation and restricts progenitor proliferation in adult homeostatic skin, with reversible effects upon inhibition (Abidi et al. 2024). Both activation and inhibition of Notch signaling alter the behaviors of cultured vascular endothelial cells, keratinocytes, and fibroblasts in a scratch wound healing model, consistent with the regulatory role of Notch signaling in the wound healing functions of these cell types (Chigurupati et al. 2007). Mechanistically, Notch signaling participates in normal wound healing by positively regulating angiogenesis, cell migration, and inflammatory responses (Kimball et al. 2017). Notch signaling, via hes family bHLH transcription factor 1 (Hes1), regulates skin aging by mitigating cellular senescence and inflammation in fibroblasts, while hes1 dysfunction in psoriasis disrupts the protein phosphatase axis, with hes1 restoration alleviating inflammation in keratinocytes (Z. Wang et al. 2022; Zou et al. 2021). Blocking Notch signaling in macrophages effectively alleviated scar formation by suppressing the inflammation and collagen deposition (He et al. 2020). Therefore, appropriate activation of the Notch pathway during skin wound healing plays a critical role in improving both the speed and quality of healing.

As a traditional Chinese medicine for relieving cough and resolving phlegm (Li et al. 2019; Zhang et al. 2020), fritillariae contains peimine, an active ingredient that exerts antitussive and anti‐inflammatory effects (Yin et al. 2019). Modulating excessive inflammatory responses appears to be a key mechanism underlying peimine's potential to promote skin wound repair. Therefore, this study aims to explore whether peimine has a pro‐healing effects on skin wound repair and its impact on healing quality. Mechanistically, peimine exerts therapeutic effects on lipopolysaccharide‐induced acute lung injury by inhibiting the nuclear factor‐kappa B (NF‐κB) signaling pathway (Liu et al. 2022). In peimine‐containing compound herbal formulations, peimine inhibits M2 macrophage polarization by promoting autophagy through suppression of the serine/threonine‐protein kinase mTOR (mTOR) signaling suppression, thereby attenuating the development of pulmonary fibrosis (Zhao et al. 2021). Furthermore, NF‐κB and mTOR signaling are downstream pathways regulated by Notch (Kong et al. 2022; Sega et al. 2019). Thus, otch signaling activation promotes wound repair, which appears to conflict with its regulatory role in inflammatory responses. Therefore, this study also investigates peimine's potential to regulate the Notch pathway during full‐thickness skin wound healing. These findings provide new insights into the molecular mechanisms by which peimine may treat full‐thickness skin wounds.

## 2 Materials and Methods

### 2.1 Experimental Animals

Nine male 8‐week‐old C57BL/6JNifdc mice were obtained from Beijing Vital River Laboratory Animal Technology Co. Ltd. The mice were housed under controlled conditions with a temperature of 22°C ± 4°C, relative humidity of 50%–60%, and a 12‐h light/dark cycle. All animals were maintained at the Animal Experimental Center of Wenzhou Medical University. The experimental protocol was approved by the Animal Ethics Committee of Wenzhou Medical University (Wenzhou, Zhejiang, China, Approval No. wydw2023‐0658) and conducted in accordance with the institutional Animal Experimentation Guidelines, ensuring humane treatment of all research animals.

### 2.2 Model Establishment and Drug Administration

All animals were anesthetized via intraperitoneal injection with 1.0% (w/v) pentobarbital sodium (40 mg · kg^−1^), followed by dorsal hair removal. Two full‐thickness skin wounds were created using an 8‐mm circular skin biopsy punch (Acuderm Co. Ltd., USA). The animals were randomly divided into three groups: the model group, the 1 mg · kg^−1^ peimine group, and the 4 mg · kg^−1^ peimine group, with three mice in each group. Peimine (#SP8140, Solarbio Co. Ltd., Beijing, China) was topically administered to the wound area.

### 2.3 Histopathological Staining

Appropriate amounts of wounds skin tissue were fixed in 4% paraformaldehyde, embedded in paraffin, and sectioned into 5‐μm‐thick slices. Hematoxylin–eosin (H&E) staining (#G1120, Solarbio) was performed to evaluate granulation tissue formation and epidermal thickness. Masson's trichrome staining (#G1346, Solarbio) was used to assess the collagen deposition in the tissue. Histological images were acquired using a Leica upright fluorescence microscope (THUNDER Imager 3D Tissue), and quantification was performed using Image Pro Plus software for semi‐quantitative evaluation of the results.

### 2.4 Immunofluorescence

Paraffin‐embedded sections of mouse skin were deparaffinized, rehydrated, and treated with citrate antigen retrieval buffer (sodium citrate buffer, 0.01 mol/L, pH 6.0, #C1010, Solarbio) at 95°C for 10 min. The slides were blocked with 5% goat serum for 1 h at 37°C and then incubated overnight at 4°C with primary antibodies: rabbit anti‐Col1α1 (1:200 dilution, #A1352, ABclonal, China), rabbit anti‐Col3α1 (1:200 dilution, #A3795, ABclonal), rabbit anti‐CD31 antibody (1:200 diluted in 1% goat serum solution, #db15306, diagbio, China), rabbit anti‐PCNA antibody (1:200 diluted in 1% goat serum solution, #db11523, diagbio), rabbit anti‐Notch1 (1:100 dilution, #bs‐1335R, Beijing Bioss, China) and mouse anti‐vimentin (1:100 dilution, #db6271, diagbio, China). The slides were washed and subsequently incubated for 1 h with fluorophore‐conjugated secondary antibodies. Secondary antibodies used in this study included CoraLite488‐conjugated goat anti‐rabbit IgG (1:100 diluted in 1% goat serum, #SA00013‐2, proteintech, China), CoraLite647‐conjugated AffiniPure F(ab′)2 Fragment Goat Anti‐Rabbit IgG (1:100 diluted in 1% goat serum, #SA00014‐9, proteintech) and CoraLite647‐conjugated AffiniPure F(ab′)2 Fragment Goat Anti‐Mouse IgG (1:100 diluted in 1% goat serum, #SA00014‐10, proteintech). Finally, nuclear counterstaining was performed and mounted with anti‐fluorescence quenching tablets containing dihydrochloride (DAPI, #36308ES20, YEASEN, China). Image acquisition and quantification used a Leica positive fluorescence microscope with consistent exposure settings across samples (THUNDER Imager 3D Tissue).

### 2.5 Cell Culture and Treatment

The NIH/3T3 cell lines (#CL‐0171, Pricella, China), a kind of fibroblast‐like cells derived from mouse embryos, were maintained in high‐glucose DMEM (4.5 g · L^−1^ D‐Glucose, C11995500BT, Gibco, USA) supplemented with 10% fetal bovine serum (#10270106, Gibco) and 1% penicillin and streptomycin mixture (#CB010, Epizyme, Shanghai, China). After starvation, NIH/3T3 cells in 6‐well plates were exposed to peimine (0, 25, and 100 μmol · L^−1^) for 24 h, then harvested for further western blot analysis.

### 2.6 Western Blot

Western blot analysis was performed to detect the protein expression levels of Notch signaling pathway‐related proteins by using rabbit anti‐Notch1 (1:1000 dilution, #bs‐1335R, Beijing Bioss), rabbit anti‐Hes1 (1:1000 dilution, #db15470, diagbio), rabbit anti‐Jagged1 (1:1000 dilution, #db14439, diagbio) and rabbit anti‐HEY1 (1:1000 dilution, #A16110, ABclonal, China). Mouse anti‐GAPDH (1:50,000 dilution, #60004‐1‐Ig, proteintech) served as a loading control. Chemiluminescent detection used Super ECL Reagent (#36208ES60, Yeasen) with ChemiDoc XRS+ System (Bio‐Rad). Band intensity quantification employed Image Lab v6.1 (Bio‐Rad), normalized to GAPDH expression.

### 2.7 Statistical Analysis

The Student's *t*‐test was used for comparisons between two groups, and one‐way ANOVA analysis was employed for comparisons among multiple groups. Graphpad Prism version 9.0 software was utilized for statistical analysis of the data. All data were expressed as mean ± standard deviation. A *p*‐value of < 0.05 was considered statistically significant.

## 3 Results

### 3.1 Peimine Accelerates Wound Healing in Mice

**FIGURE 1:**
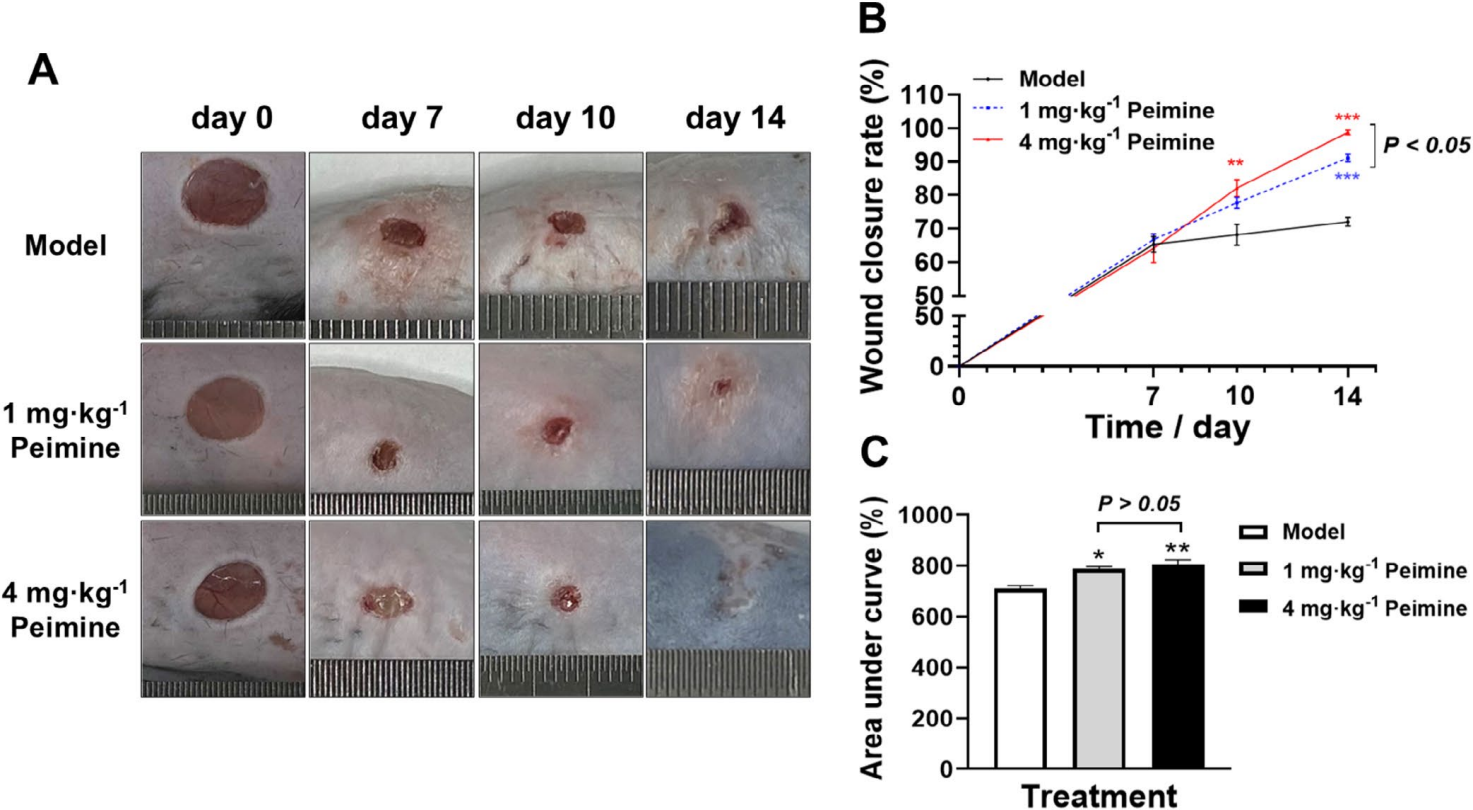
Wound healing in mouse skin. (A) Skin wounds in mice (scale bar: 15 mm, *n* = 3 per time point and group). (B) Wound healing rate. (C) Area under the curve of healing rates (*n* = 3). Compared with the model group: *p < 0.05, **p < 0.01, ***p < 0.001.

Wound integrity serves as a crucial indicator for assessing the rate of wound healing. In this study, to evaluate the potential of peimine in enhancing skin wound healing in mice, Figure 1A displays a series of representative wound images captured on days 0, 7, 10, and 14 during different treatment periods. As illustrated in Figure 1B, the peimine‐treated group exhibited significantly accelerated wound closure rates in C57BL/6 mice on days 10 and 14 compared to the model group (*p* < 0.01 or *p* < 0.001), but not on day 7 (*p* > 0.05). Specifically, by day 14, the 4 mg · kg^−1^ peimine group achieved a 98.72% ± 1.203% closure rate, outperforming both the 1 mg · kg^−1^ group (91.12% ± 2.000%) and the model group (72.03% ± 2.265%, *p* < 0.05). However, on days 7 and 10, there was no significant difference in wound closure rate between the groups receiving a 1 and 4 mg · kg^−1^ dose of peimine (*p* > 0.05).

Area under the curve (AUC) analysis of healing rates further confirmed dose‐dependent efficacy. In detail, compared to the model group, analysis of the AUC for healing rates revealed that both 1 and 4 mg · kg^−1^ peimine‐treated groups significantly promoted wound closure rates (Figure 1C, *p* < 0.05 and *p* < 0.01, 788.1% ± 13.83% and 805.5% ± 30.49% vs. 709.8% ± 20.94%). In addition, the 4 mg · kg^−1^ group showed a 7.87% higher AUC than the 1 mg · kg^−1^ group; this difference lacked statistical significance (Figure 1C, *p* > 0.05).

### 3.2 Peimine Enhances Granulation Tissue Formation and Epithelial Regeneration

**FIGURE 2:**
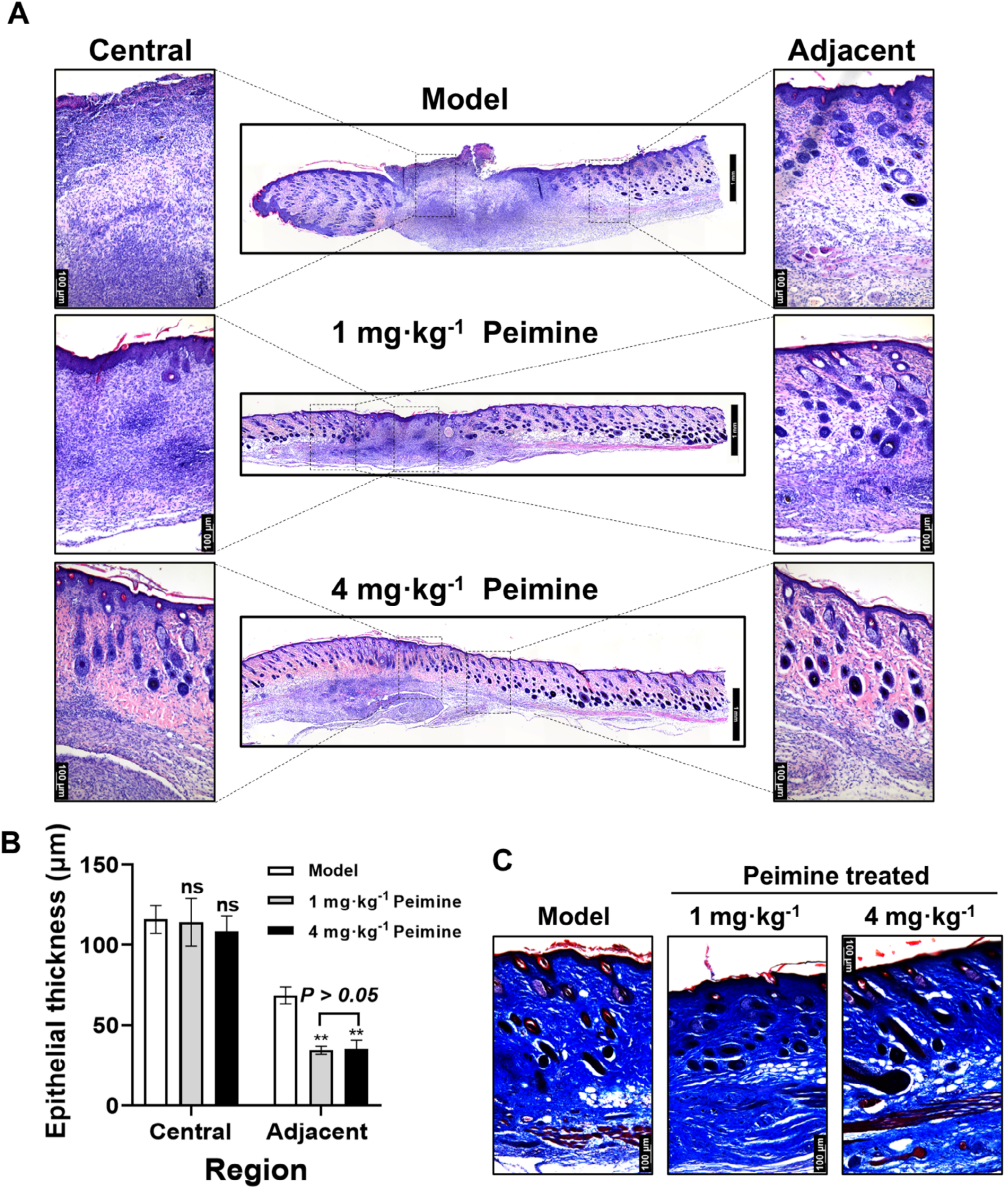
Granulation tissue formation and epithelial regeneration in mouse skin. (A) H&E staining of full thickness of skin from central and adjacent regions of the wound on day 14. (B) Statistical analysis of epidermal thickness in central and adjacent wound regions (*n* = 3). (C) Masson's trichrome staining of skin on day 14. Compared with the model group: ns, not significant; **p < 0.01.

Moreover, to evaluate the improvement in granulation tissue formation in the peimine‐treated groups, histological analysis was conducted using H&E staining on skin tissues from each group on day 14. High‐quality wound healing includes intact epithelium, mature dermis, and orderly arrangement of ECM without obvious inflammatory lesions (Bi et al. 2023; J. Wang et al. 2021; Zhu et al. 2024). Histological analysis via H&E staining demonstrated improved tissue architecture in peimine‐treated groups (Figure 2A). Analysis by H&E staining results, compared to the model group, the 4 mg · kg^−1^ peimine‐treated group displayed intact epithelium with mature dermal collagen alignment, hair follicle regeneration, and reduced inflammation. In contrast, the 1 mg · kg^−1^ peimine‐treated group still exhibited partial dermal defects, while the model group exhibited epithelial discontinuity and persistent inflammation.

To assess the effect of peimine on epithelial regeneration during wound healing, the thickness of the epithelium was measured on day 14. In the central area of the wound, the epithelial thickness of the skin was similar in each group with no significant differences (Figure 2B, *p* > 0.05). Unexpectedly, the epithelial thickness adjacent to the wound in both the 1 and 4 mg · kg^−1^ peimine‐treated groups showed significantly thinner epidermal layers compared to the model group (*p* < 0.01; 34.37 ± 4.329 μm and 35.11 ± 9.746 μm vs. 68.58 ± 9.198 μm). Furthermore, the epithelial thickness of the 4 mg · kg^−1^ peimine‐treated group did not show a significant difference compared to the 1 mg · kg^−1^ peimine‐treated group (*p* > 0.05). Overall, our results demonstrated that peimine treatment effectively promotes epithelial regeneration and maturation, contributing to faster wound healing.

### 3.3 Peimine Significantly Promotes Collagen Deposition in Wound Tissue

**FIGURE 3:**
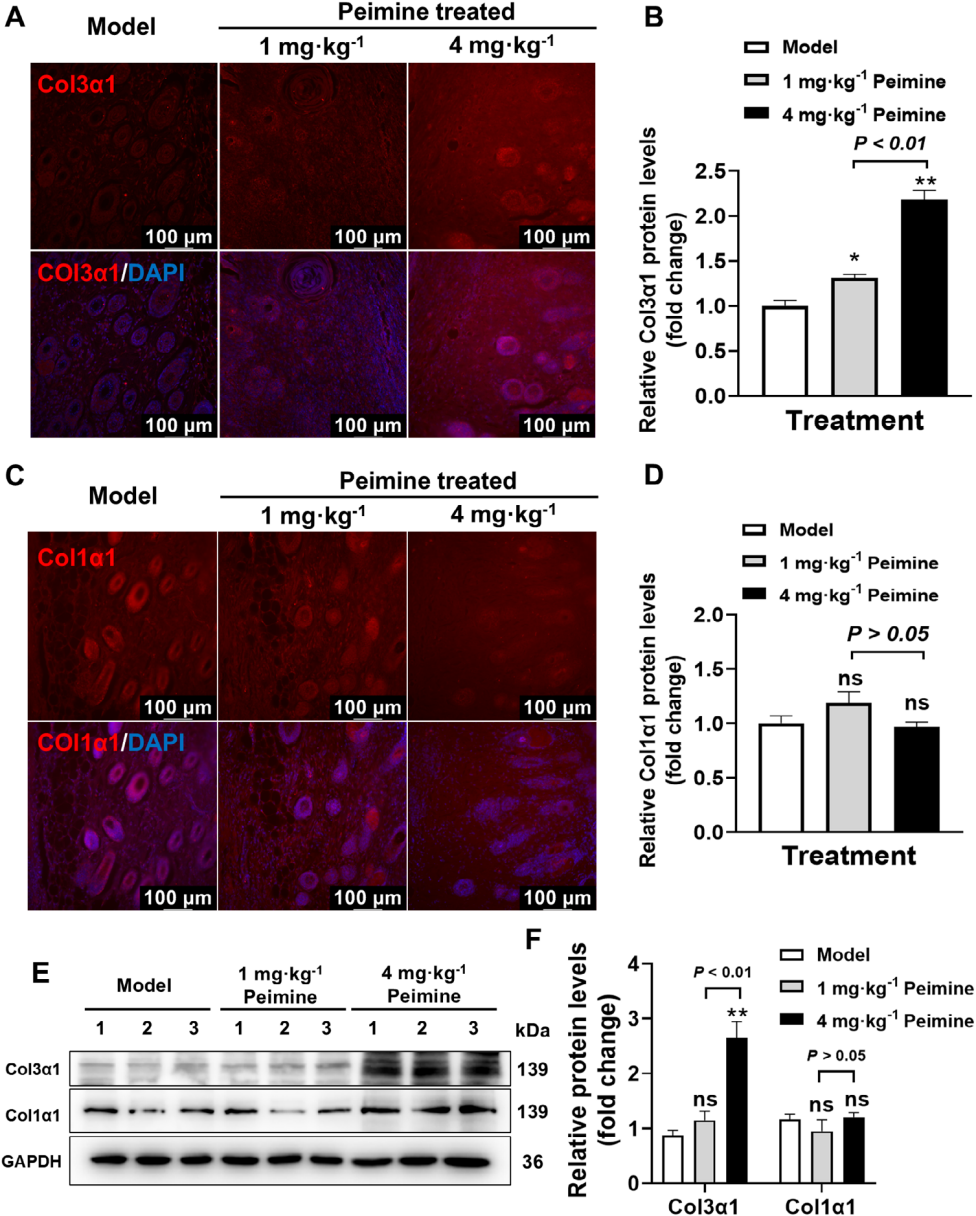
Promoted collagen expression. (A) Immunofluorescence (IF) of Col3α1 in the dermis. (B) Statistical analysis of Col3α1 measured by IF in the dermis (*n* = 3). (C) IF of Col1α1 in the dermis. (D) Statistical analysis of Col1α1 measured by IF in the dermis (*n* = 3). (E,F) Western blot results (E) and their statistical analysis (F) of Col3α1 and Col1α1 protein expression levels (*n* = 3). Compared with the model group: ns, not significant; *p < 0.05, **p < 0.01.

Collagen, as the primary component of the ECM, provides structural support to tissues and plays a crucial role in wound healing. Masson's trichrome staining revealed that peimine significantly enhanced the deposition of total collagen, as indicated by deeper blue staining, in the skin tissue of the wound area (Figure 2C). Since Masson's trichrome staining cannot fully reveal the quantity of ECM deposition and expression in the skin, we focused on examining the protein expression of type I and III collagens, which play vital roles in the formation of new ECM. IF staining was used to assess the levels of Col1α1 and Col3α1 proteins in the dermis. As shown in Figure 3A,B, compared to the model group, Col3α1 protein levels were significantly increased in the peimine‐treated groups on day 14 (*p* < 0.05 or *p* < 0.01). Moreover, the 4 mg · kg^−1^ peimine‐treated group showed a significant increase in Col3α1 protein expression compared to the 1 mg · kg^−1^ peimine‐treated group (*p* < 0.01). However, Col1α1 protein levels in each group did not show significant differences (Figure 3C,D, *p* > 0.05). Western blot results (Figure 3E,F) further demonstrated that the expression level of Col3α1 protein was significantly increased in the 4 mg · kg^−1^ peimine‐treated group compared to both the model group and the 1 mg · kg^−1^ peimine‐treated group (*p* < 0.01). In conclusion, our results demonstrated that peimine treatment effectively promotes the expression of Col3α1 during skin wound healing in mice.

### 3.4 Peimine Significantly Promotes Cell Proliferation and Angiogenesis

**FIGURE 4:**
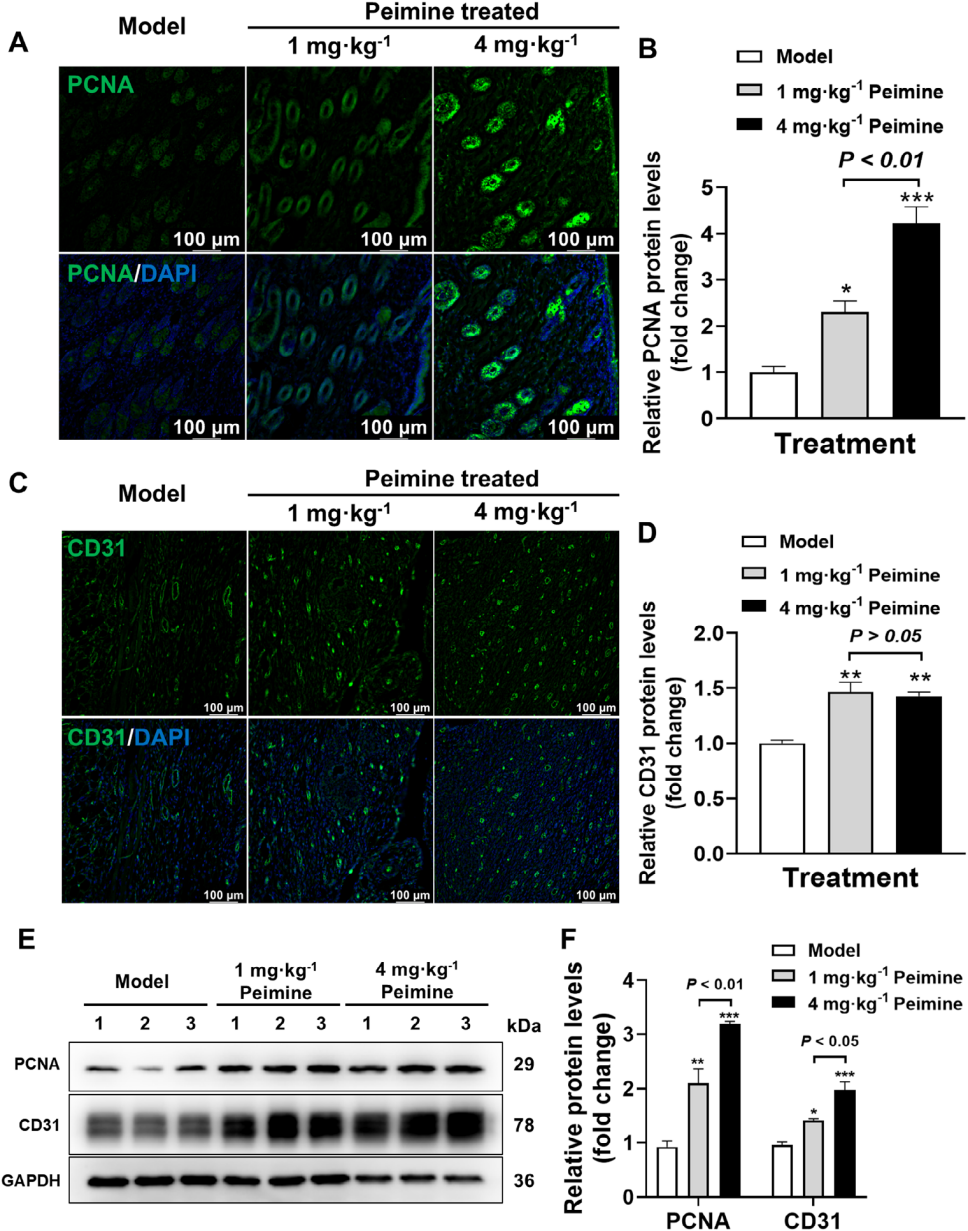
Promoted proliferation and angiogenesis. (A) IF of PCNA in the dermis. (B) Statistical analysis of PCNA measured by IF in the dermis (*n* = 3). (C) IF of CD31 in the dermis. (D) Statistical analysis of CD31 measured by IF in the dermis (*n* = 3). (E, F) Western blot results (E) and their statistical analysis (F) of PCNA and CD31 protein expression levels (*n* = 3). Compared with the model group: *p < 0.05, **p < 0.01, ***p < 0.001.

Proliferating cell nuclear antigen (PCNA) is an essential factor in DNA replication and repair (González‐Magaña and Blanco 2020), which is commonly used as a proliferative indicator in the evaluation of skin healing (Yang et al. 2020). To evaluate the proliferative effects, the expression of PCNA in the dermis was assessed on day 14. IF staining revealed significantly higher PCNA expression in the 1 and 4 mg · kg^−1^ peimine‐treated groups compared to the model group (2.307‐ and 4.217‐fold, respectively, *p* < 0.05 and *p* < 0.01, Figure 4A,B). Additionally, the expression level of PCNA protein is significantly increased in the 4 mg · kg^−1^ peimine‐treated group compared with the 1 mg · kg^−1^ peimine‐treated group (*p* < 0.01).

In the process of skin healing, blood vessel regeneration is a crucial step. As a marker of endothelial cells, the high expression of CD31 is closely related to the formation of new blood vessels and helps to accelerate the skin healing process (Liang et al. 2019). IF staining for CD31 demonstrated that peimine significantly promoted angiogenesis in the skin wound, leading to a significant increase in CD31 positive staining in the 1 and 4 mg · kg^−1^ peimine‐treated groups compared to the model group (1.465‐ and 1.428‐fold, respectively, *p* < 0.01, Figure 4C,D). However, compared with the 1 mg · kg^−1^ peimine‐treated group, the 4 mg · kg^−1^ peimine‐treated group exhibited no statistically significant difference in the expression of CD31 protein (*p* > 0.05). Western blot results (Figure 4E,F) showed that the expression level of PCNA and CD31 protein was significantly increased in both the 1 and 4 mg · kg^−1^ peimine‐treated groups compared with the model group (*p* < 0.01 or *p* < 0.001). In summary, our results confirmed that peimine treatment significantly promotes the expression levels of the proliferative biomarker PCNA and angiogenesis biomarker CD31 during skin wound healing in mice.

### 3.5 Peimine Significantly Activates the Notch1 Signaling Pathway

**FIGURE 5:**
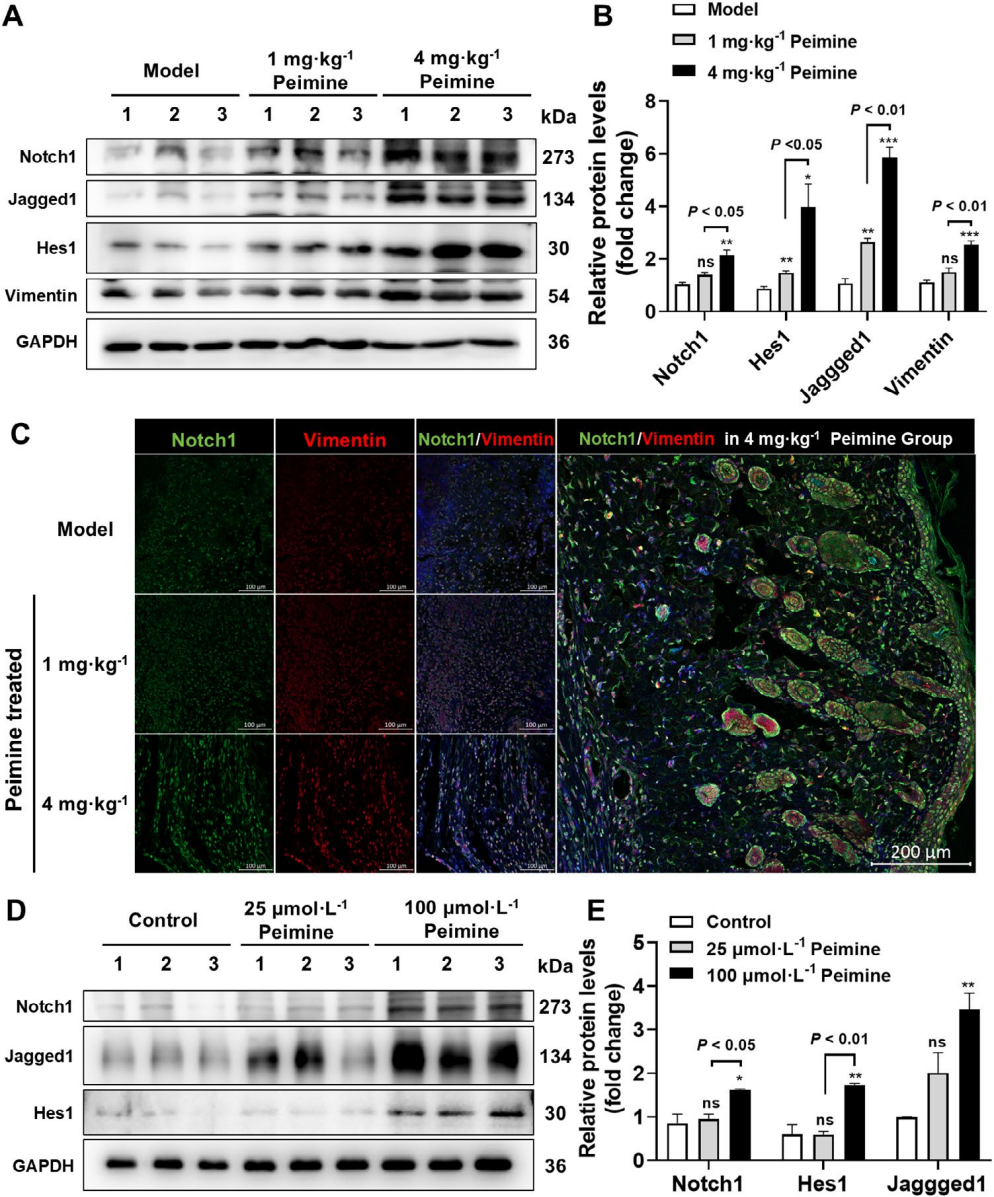
Activited Notch1 signaling pathway. (A) Western blot of Notch1, Hes1, Jagged1, and vimentin in wound sections on days 14. (B) Statistical analysis of the above protein measured by Western blot (*n* = 3). (C) Co‐immunofluorescence staining of Notch1 (Green) and vimentin (Red) of wound sections in mice on days 14. (D, E) Western blot results (D) and their statistical analysis (E) of protein expression levels of Notch1, Hes1 and Jagged1 in NIH/3T3 cells after peimine treatment for 24 h (*n* = 3). Compared with the model or control group, respectively: ns, not significant; *p < 0.05, **p < 0.01, ***p < 0.001.

In this study, we aimed to investigate whether peimine exerts its wound healing effects through the Notch1 signaling pathway. Therefore, we examined the expression levels of proteins associated with the Notch1 signaling pathway in the wound tissue. As shown in Figure 5A,B, compared to the model group, the 4 mg · kg^−1^ peimine‐treated group significantly upregulated the protein expression levels of Notch1 (*p* < 0.01). The 4 mg · kg^−1^ peimine‐treated group exhibited an overall improvement of 54.23% in Notch1 protein expression compared to the group receiving a 1 mg · kg^−1^ dose (Figure 5B, *p* < 0.05). In addition, the protein expression levels of Hes1, Jagged1, and Hey1—biomarkers of downstream transcription proteins activated by Notch1 signaling pathways—were all significantly upregulated in the 1 and 4 mg · kg^−1^ peimine‐treated groups compared to the model group in the mouse wound (*p* < 0.05). These results all confirmed that peimine treatment significantly promotes activation of the Notch1 signaling pathway.

Activation of the Notch1 signaling pathway in fibroblasts has been reported to improve their proliferation and myofibroblastic function (Hsu et al. 2017; Huo et al. 2023). Therefore, the expression of vimentin, a fibroblast biomarker in the dermis of mouse skin wounds, was determined by western blot. The results confirmed that the protein expression level of vimentin was significantly increased in the 4 mg · kg^−1^ peimine‐treated groups compared to the model group in the mice wounds (Figure 5B, *p* < 0.001). In order to explore the activation of Notch1 in fibroblasts of skin in peimine‐treated groups, IF results demonstrated that Notch1 was mainly expressed and co‐localized with vimentin (Figure 5C). Thus, it was hypothesized that peimine might upregulate Notch1 expression and activate related signaling pathways in skin fibroblasts. So, in the fibroblast cell line NIH‐3T3, western blot results showed the expression level of Notch1, Hes1, and Jagged1 proteins were all significantly increased in the 100 μmol · L^−1^ peimine‐treated group compared with the control group (Figure 5D,E, *p* < 0.05 or *p* < 0.01), whereas the 25 μmol · L^−1^ peimine treatment had no significant effect (*p* > 0.05). Taken together, peimine could activate the Notch1 signaling pathway in skin fibroblasts, which might contribute to faster and higher‐quality wound healing.

## 4 Discussion

The evaluation of wound healing primarily hinges on several key metrics, including the rate of wound healing, ECM deposition, proliferation, angiogenesis, and the control of the inflammatory response (Peña and Martin 2024). The high quality of wound healing refers to the degree and effectiveness of the healing process, encompassing crucial factors such as healing speed, scar appearance, and the recovery of normal skin function (Jeschke et al. 2023). It was reported that peimine exhibits anti‐inflammatory effects in the treatment of osteoarthritis and asthma, primarily by inhibiting inflammatory factors such as interleukin‐1β, NF‐κB, and tumor necrosis factor α‐mediated inflammation via the mitogen‐activated protein kinase (MAPK) pathway, thereby playing a therapeutic role (Chen et al. 2019; Peng et al. 2023; Zhou et al. 2022). Notably, the MAPK pathway not only regulates inflammation but also governs a wide array of important cellular physiological and pathological processes, including cell growth and differentiation (Scepanovic et al. 2021; Sun et al. 2023). This mechanism prompts us to investigate whether peimine can facilitate skin wound healing by regulating the proliferation process during the healing phase. In this study, we comprehensively demonstrated that treatment with 1 and 4 mg · kg^−1^ of peimine significantly promotes skin wound repair in mice from multiple aspects.

The size of the skin wound and the integrity of the epidermis are important indices for evaluating the rate of skin healing. Our results confirmed that, in terms of wound healing, both 1 and 4 mg · kg^−1^ of peimine significantly reduced the wound area and accelerated the healing process on days 10 and 14 (Figure 1). However, with regard to dosage, the 4 mg · kg^−1^ peimine treatment demonstrated only a slight advantage in promoting wound healing on day 14, and the AUC did not exhibit a statistically significant difference. Therefore, future studies should explore higher doses to amplify therapeutic differences. Additionally, on day 14, there were still notable unhealed areas on the wound surface of the model group, which compromise the integrity of the skin. From a superficial perspective, our results confirm that peimine therapy promotes wound repair.

In the healing process, healthy granulation tissue, composed of neovascularization, fibroblasts, and ECM filling the wound bed, serves as a scaffold for tissue regeneration and provides a foundation for re‐epithelialization. Staining results from pathological sections showed that although the 1 mg · kg^−1^ peimine treatment group had complete epithelium, the dermis was still immature, with persistent inflammation and absence of hair follicles. During the wound healing process, collagen is synthesized and organized to restore skin integrity (Gajbhiye and Wairkar 2022; Jeschke et al. 2023). Correct collagen synthesis and remodeling are crucial for achieving strong and flexible scars, thereby improving the quality of healing. Col1α1 and Col3α1 play distinct physiological roles in the strength and elasticity of skin during late‐stage wound healing. However, in our study, peimine significantly promoted the expression of Col3α1 proteins (Figure 3). These findings confirm that peimine treatment significantly increases specific collagen deposition in the dermis. Moreover, proliferation and angiogenesis are critical processes in skin wound healing, essential for successful tissue repair (Shou et al. 2023). Proliferation is responsible for the rapid growth and multiplication of cells during wound healing, while angiogenesis involves the formation of new blood vessels, crucial for supplying oxygen, nutrients, and immune cells to the wound. Our results demonstrate that peimine administration significantly increases the expression of PCNA in both the epidermis and dermis (Figure 4). The vascular marker CD31 is predominantly distributed in the dermis, and peimine treatment markedly enhances angiogenesis (Figure 4). A limitation of this study is the lack of investigation into peimine's regulation of inflammatory responses during wound healing, which warrants future research.

This study contributes the discovery that peimine potentially promotes wound healing by upregulating proteins in the Notch1 signal pathway (Figure 5). Proliferating cells during wound healing include fibroblasts and keratinocytes (Y. C. Wang et al. 2023). Here, we confirmed the co‐localization of Notch1 with vimentin‐positive fibroblasts and Notch1 pathway activation by peimine in vitro using cell lines. However, keratinocytes are pivotal for maintaining skin epithelial integrity and function (Dekoninck and Blanpain 2019; Headon 2017; Y. C. Wang et al. 2023). Immunofluorescence staining revealed Notch1 expression in the epithelium and peri‐follicle regions, suggesting keratinocytes as potential targets of peimine. The specific mechanism remains unclear, necessitating future studies to identify keratinocyte subtypes regulated by peimine via the Notch1 pathway.

## 5 Conclusion

This study demonstrates that peimine significantly accelerates skin wound repair in mice through Notch1 signaling pathway activation in fibroblasts, as evidenced by upregulated PCNA and CD31 expression, enhanced Col3α1 deposition, and improved granulation tissue maturation. Mechanistically, peimine promotes epithelial regeneration, collagen remodeling, and angiogenesis via Notch1‐dependent regulation of downstream targets (Hes1, Jagged1, Hey1) both in vivo and in NIH/3T3 fibroblasts. However, limitations include: (1) incomplete characterization of peimine's anti‐inflammatory effects during early healing stages; (2) unclear dose‐dependent efficacy beyond 4 mg · kg^−1^; and (3) unresolved crosstalk between Notch1 and keratinocytes, despite observed peri‐follicular Notch1 expression. Future studies should prioritize: (1) optimizing dosing regimens to balance efficacy and safety; (2) defining Notch1's role in keratinocyte differentiation using lineage‐specific knockouts; and (3) validating findings in human ex vivo or 3D skin models. These insights not only advance peimine's translational potential as a multi‐target wound therapeutic but also highlight Notch1 signaling as a pivotal node for high‐quality skin regeneration.

## Author Contributions

**Peng Wu:** conceptualization (equal), formal analysis (equal), investigation (equal), writing – original draft (equal), writing – review and editing (equal). **Cheng Zhang:** data curation (equal), methodology (equal), writing – original draft (equal), writing – review and editing (equal). **Congcong Wu:** investigation (equal), methodology (equal), visualization (equal). **Junzhe Lang:** investigation (equal), methodology (equal). **Lili He:** conceptualization (lead), project administration (lead), resources (lead), supervision (lead), writing – review and editing (lead).

## Ethics Statement

This study was approved by the Animal Ethics Committee of Wenzhou Medical University (Ethical Approval No. wydw2023‐0658).

## Conflicts of Interest

The authors declare no conflicts of interest.

## Data Availability

Data available on request from the authors.
